# Retraction: Optimising barrier placement for intrusion detection and prevention in WSNs

**DOI:** 10.1371/journal.pone.0351198

**Published:** 2026-06-23

**Authors:** The PLOS One Editors

The *PLOS One* Editors retract this article [1] due to concerns about compromised peer review.

The Editors have no evidence of any author involvement in the peer review concerns.

All authors did not agree with the retraction.
